# Association of Serum Angiopoietin-Like Protein 8 With Albuminuria in Type 2 Diabetic Patients: Results From the GDMD Study in China

**DOI:** 10.3389/fendo.2018.00414

**Published:** 2018-07-18

**Authors:** Liuxue Yang, Jianfei Song, Xiaoxi Zhang, Liuping Xiao, Xueping Hu, Haidong Pan, Linyuan Qin, Hongbo Liu, Bo Ge, Tianpeng Zheng

**Affiliations:** ^1^Department of Endocrinology and Metabolism, The Second Affiliated Hospital of Guilin Medical University, Guilin, China; ^2^Department of Thoracic and Cardiovascular Surgery, The Second Affiliated Hospital of Guilin Medical University, Guilin, China; ^3^Center of Diabetic Systems Medicine, Guilin Medical University, Guilin, China; ^4^Department of Epidemiology and Health Statistics, Guilin Medical University, Guilin, China; ^5^Department of Laboratory Medicine, The Second Affiliated Hospital of Guilin Medical University, Guilin, China; ^6^Department of Urology, The Second Affiliated Hospital of Guilin Medical University, Guilin, China

**Keywords:** ANGPTL8, triglycerides, albuminuria, diabetic kidney disease, type 2 diabetes

## Abstract

**Background:** Hyperglycemia, insulin resistance and hypertriglyceridesmia are risk factors for albuminuria in type 2 diabetes. Angiopoietin-like Protein 8(ANGPTL8) is a newly identified liver-derived hormone related to these risk factors. Hence, we aimed to explore the relationship between ANGPTL8 and albuminuria in type 2 diabetes.

**Methods:** Serum ANGPTL8 levels were determined in groups of control (*n* = 50) and type 2 diabetic patients with normoalbuminuria (A1, *n* = 100), microalbuminuria (A2, *n* = 45), and macroalbuminuria (A3, *n* = 33).

**Results:** Serum levels of ANGPTL8 and triglycerides were significantly increased in type 2 diabetic patients with albuminuria as compared with controls (*P* < 0.001). ANGPTL8 levels were positively correlated with triglycerides, duration of diabetes, and urine albumin-to-creatinine ratio (ACR) and negatively correlated with estimated glomerular filtration rate in type 2 diabetic patients with A2 and A3 (all *P* < 0.05). Logistic regression analysis indicated that ANGPTL8 had higher odds of having A2 (*OR* = 2.52, 95% CI 1.16–5.48, *P* = 0.019) and A3 (*OR* = 4.89, 95% CI 2.10–11.39, *P* < 0.001) in type 2 diabetes. Mediation analysis indicated that triglycerides might act as a partial mediator in the relationship between ANGPTL8 and ACR.

**Conclusions:** Triglycerides might partially mediate the correlation between ANGPTL8 and ACR. Our data provide the evidence for a strong link between ANGPTL8 and albuminuria, indicating that ANGPTL8 may be a new biomarker for diabetic kidney disease in type 2 diabetes.

Trial Registration Number: ChiCTR-EPC-14005273

## Introduction

Diabetic kidney disease is one of the most common microvascular complications of diabetes, which results in chronic kidney disease in diabetic patients. It is one of the important causes of endstage renal disease and cardiovascular mortality in developed and developing countries (1, 2). The underlying mechanism of diabetic kidney disease is still not entirely clear, therefore, it is of great important to identify novel biomarkers and detailed pathogenesis linked to diabetic kidney disease.

Angiopoietin-like protein 8 (ANGPTL8), also known as betatrophin, lipasin, TD26 and refeeding-induced fat and liver (RIFL), is a secreted protein of 198 amino acids primarily expressed in liver and adipose tissues (3). It is a newly identified endocrine regulator associated with insulin resistance, lipid and glucose metabolism, all of which are thought to be involved in the pathogenesis of diabetic kidney disease in type 2 diabetes (4). Animal studies showed that ANGPTL8 was positively related to triglycerides (TG) levels, TG levels were significantly lower in mice lacking ANGPTL8 than in control mice (5), whereas an overexpression of ANGPTL8 led to an increase in TG levels (6). A previous cohort study with the largest sample size has proved that circulating levels of ANGPTL8 were significantly higher in type 2 diabetic patients than in control subjects with normal glucose tolerance (7), in addition, other studies and meta-analysis have also shown that ANGPTL8 was increased in people with type 2 diabetes (8–15). Furthermore, some studies have reported a positive relationship between blood glucose, homeostasis model assessment of insulin resistance (HOMA-IR) and ANGPTL8 in type 2 diabetic patients (10, 11). Consequently, these findings above motivated us to speculate that increased circulating levels of ANGPTL8 might be positively related to urine albumin-to- creatinine ratio (ACR) and serve as a novel biomarker for diabetic kidney disease in type 2 diabetes.

Thus, the aim of our study was to answer three questions: (1) Whether serum levels of ANGPTL8 are increased in type 2 diabetic patients with albuminuria. (2) Whether increased levels of ANGPTL8 are positively related to albuminuria in type 2 diabetic patients. (3) If the second answer is yes, what factors might mediate the relationship between ANGPTL8 and albuminuria in type 2 diabetes?

## Materials and methods

### Study subjects

This was a cross-sectional analysis of baseline data from Guangxi Diabetes and Metabolic Disorders (GDMD) study aimed to investigate the etiology and comorbidities of type 2 diabetes and metabolic syndrome (16). From 2015 to 2016, a total of 228 subjects attending Medical Examination Center of the Affiliated Hospital of Guilin Medical University were selected to participate in this study, the subjects were then classified into four groups: (1) control subjects with normal glucose tolerance and normoalbuminuria (A1, ACR < 30 mg/g, *n* = 50); (2) type 2 diabetic patients with A1 (*n* = 100); (3) type 2 diabetic patients with microalbuminuria (A2, ACR between 30 and 300 mg/g, *n* = 45); (4) type 2 diabetic patients with macroalbuminuria (A3, ACR > 300 mg/g, *n* = 33). Exclusion criteria were as follows: (1) Control subjects taking any medication known to affect insulin sensitivity, glucose tolerance and lipid metabolism. (2) Angiotensin-converting enzyme inhibitors, angiotensin receptor blocker or insulin use. (3) Use of possible or known drugs affecting TG for more than 3 months or at any time within 6 months before the recruitment. (4) Any evidence of acute inflammatory diseases, acute diabetic complications, liver, heart, kidney and respiratory failure, malignancy. (5) Participants without complete data. The study was approved by the Ethics Committee of Affiliated Hospital of Guilin Medical University, and written informed consent has been obtained from each participant after full explanation of the purpose and nature of our study. This study was registered on the Chinese Clinical Trial Registry (ChiCTR-EPC-14005273).

### Measurements

A standard questionnaire was administered to all subjects to record demographic and clinical data. Anthropometric parameters were measured as previously described (17). Participants were instructed to maintain usual physical activity and diet for at least 3 days before the examination. After an overnight fast of 10 h or longer, blood samples were collected to measure blood lipids, HbA1c, fasting insulin, fasting plasma glucose (FPG), and ANGPTL8, blood samples were also drawn at 120 min after a 75 g glucose load to measure glucose in controls subjects. Additionally, two successive early morning urine samples were collected to measure average ACR. Serum levels of ANGPTL8 were quantified using a commercially available ELISA kit (Wuhan Eiaab Science, Wuhan, China) according to the manufacturer's instructions. The measurement of ACR was performed as previously described (18). We calculated estimated glomerular filtration rate (eGFR) using the CKD-EPI equation. The CKD-EPI equation was expressed as GFR = 141 × min(SCr/*k*,1)^α^ × max(SCr/*k*,1)^−1.209^ × 0.993^age^ × 1.018 (if female), where *k* is 0.7 for females and 0.9 for male, α is −0.329 for females and −0.411 for males—min indicates the minimum of SCr/*k* or 1, and max indicates the maximum of SCr/*k* or 1. The homeostasis model assessment of insulin resistance (HOMA-IR) was calculated as previously reported (19).

### Statistical analysis

Statistical analyses were conducted using SPSS 16.0 software. Continuous variables were described as means ± standard deviation, whereas skewed variables (fasting insulin, TG, HOMA-IR and urinary ACR) were log-transformed before statistical analysis. ANOVA and chi-square were performed to compare clinical and biochemical parameters. Correlation analysis and multivariate linear regressions were used to examine the relationship between the investigated variables. Binary logistic regression analysis was performed to estimate the association of duration of diabetes, TG and ANGPTL8 with the odds of albuminuria in type 2 diabetes.

To determine whether the association between ANGPTL8 and ACR was mediated by hyperglycemia, TG, or insulin resistance, mediation analysis was conducted based on the procedures outlined by Baron and Kenny (20). A three-step linear regression model was constructed as follows: (1) *Y* = *cX* + e1 (2) *M* = *aX* + e2 (3) *Y* = *c*′*X* + b*M* + e3, where X is the independent variable (ANGPTL8), Y is the dependent variable (ACR), M is the mediator, a is the regression coefficient for the association between ANGPTL8 and mediator, b is the regression coefficient for the association between mediator and ACR, c is the regression coefficient for the association between ANGPTL8 and ACR, and c′ is the effect of ANGPTL8 on ACR while controlling for the indirect effect. An indirect ratio was used to present the strength of mediation: ([a^*^b]/*c*). In this analysis, four conditions used to establish mediation were as follows: (1) the independent variable should be significantly associated with the dependent variable; (2) the independent variable should be significantly associated with the mediator; (3) the mediator should be significantly associated with the dependent variable; (4) the relationship between the dependent and independent variable should be attenuated when the mediator is included in the regression model.

## Results

### Clinical and laboratory characteristics

The detailed clinical characteristics of the four groups were presented in Table 1. No significant differences in age, sex, BMI, current smoking, habitual alcohol drinking, total cholesterol (TC), low-density lipoprotein cholesterol (LDL-C) and high-density lipoprotein cholesterol (HDL-C) levels between the three diabetic groups and the control group were observed. Significant differences were found in statin use, NSAID use, systolic blood pressure (SBP), diastolic blood pressure (DBP), TG, fasting plasma glucose, fasting insulin, HOMA-IR, duration of diabetes, HbA1c, eGFR and urinary ACR between the diabetic patients and the control subjects (all *P* < 0.05). We found that serum levels of ANGPTL8 were significantly increased in type 2 diabetic patients with A1, A2, and A3 compared with control subjects (*P* < 0.001).

**Table 1:** Clinical characteristics in control subjects with normal glucose tolerance (NGT), and T2DM patients with A1, A2, and A3.

**Characteristics**	**Control subjects with NGT**	**Type 2 diabetic patients**			***P*-value**
		**A1**	**A2**	**A3**	
N	50	100	45	33	–
Age (years)	56.5 ± 7.0	55.4 ± 8.3	54.2 ± 7.5	56.8 ± 8.5	0.403
Percent men (%)	60.0	51.0	44.4	60.6	0.357
Body mass index (kg/m^2^)	24.8 ± 3.6	25.0 ± 3.2	24.4 ± 3.5	24.7 ± 3.7	0.777
Current smoking (%)	22	27	33.3	24.2	0.641
Habitual alcohol drinking (%)	26	20	28.9	24.2	0.664
Leisure-time physical activity (%)	66	48	37.8	42.4	0.034
Statin use (%)	0	38	37.8	48.5	<0.001
NSAID use (%)	0	28	46.7	36.4	<0.001
SBP^a^	126 ± 8	135 ± 17	134 ± 14	144 ± 15	<0.001
DBP^a^	71 ± 8	75 ± 11	74 ± 11	79 ± 10	0.011
TG (mmol/L)^a^	1.22 (0.80, 1.67)	1.55 (1.10, 2.49)	2.07 (1.48, 3.99)	3.39 (1.60, 4.25)	<0.001
TC (mmol/L)^a^	4.79 ± 0.81	5.10 ± 0.85	4.87 ± 1.06	5.01 ± 1.32	0.312
LDL-C (mmol/L)^a^	2.84 ± 0.73	3.15 ± 1.10	3.11 ± 0.84	3.29 ± 1.16	0.185
HDL-C (mmol/L)^a^	1.28 ± 0.37	1.17 ± 0.41	1.13 ± 0.30	1.23 ± 0.43	0.203
Fasting plasma glucose (mmol/L)	4.85 ± 0.62	8.59 ± 1.66	8.10 ± 1.32	8.14 ± 1.94	<0.001
Fasting insulin (μU/ml)	6.85 (5.44, 10.21)	9.03 (7.41, 12.46)	11.08 (6.37, 21.20)	11.76 (7.08, 16.00)	<0.001
HOMA-IR	1.59 (1.07, 2.09)	3.48 (2.72, 5.00)	3.79 (2.28, 7.93)	3.70 (2.43, 5.72)	<0.001
Duration of diabetes (years)	–	6.5 ± 2.9	8.0 ± 4.7	8.5 ± 5.0	<0.001
HbA1c (%)^a^	5.0 ± 0.4	7.7 ± 1.3	8.0 ± 1.2	7.6 ± 0.9	<0.001
HbA1c (mmol/mol)^a^	31.8 ± 4.8	61.1 ± 14.3	64.5 ± 13.7	59.9 ± 9.7	<0.001
eGFR (mL/min/1.73 m ^2^)^a^	90.6 ± 21.4	81.0 ± 20.3	77.0 ± 21.8	40.2 ± 16.8	<0.001
Urinary ACR (mg/g)^a^	5.6 (2.4, 9.4)	9.5 (5.7, 18.3)	103.5 (75.0, 191.7)	895.0 (650.6, 1,191.5)	<0.001
ANGPTL8^a^ (pg/mL)	803 ± 410	1,297 ± 354	1,506 ± 669	1,755 ± 848	<0.001

*Data were expressed as means ± standard deviation, median (interquartile range), or percentage for continuous various and categorical variables, respectively. Cigarette smoking was defined as having smoked at least 100 cigarettes in one's lifetime. Regular leisure-time physical activity was defined as participation in ≥30 min of moderate or vigorous activity per day at least 3 days per week*.

a *Adjusted for age, gender, and BMI*.

### Correlation analysis between ANGPTL8 and other variables

Partial correlation analysis for ANGPTL8 and other parameters in four groups were listed in Table 2. Serum ANGPTL8 levels positively correlated with duration of diabetes in all type 2 diabetic groups (all *P* < 0.05). In subgroups of type 2 diabetic patients categorized by albuminuria, serum ANGPTL8 levels were positively correlated with TG and ACR and negatively correlated with eGFR in the subgroups of type 2 diabetic patients with A2 and A3, significant correlation was not observed between ANGPTL8 and TG, ACR or eGFR in the subgroups of type 2 diabetic patients with A1. Multivariate linear regression analysis controlling for age, sex and BMI further revealed that duration of diabetes, TG, eGFR and ACR were independently related to serum levels of ANGPTL8 in the subgroups of type 2 diabetic patients with A2 and A3 (all *P* < 0.05; Table 3).

**Table 2:** Correlations analysis of variables with circulating ANGPTL8 levels in control subjects with normal glucose tolerance (NGT), and T2DM patients with A1, A2, and A3.

	**Control subjects with NGT**		**Type 2 diabetic patients**					
			**A1**		**A2**		**A3**	
	***r***	***p***	***r***	***p***	***r***	***p***	***r***	***p***
Age	0.001	0.994	0.139	0.167	0.046	0.766	0.023	0.898
BMI	−0.002	0.988	−0.112	0.268	0.101	0.508	0.309	0.080
SBP^a^	0.134	0.370	−0.111	0.280	−0.013	0.937	0.168	0.374
DBP^a^	0.146	0.327	−0.106	0.303	0.011	0.943	0.085	0.654
TG^a^	0.158	0.288	0.191	0.061	0.379	0.013	0.381	0.038
TC^a^	0.127	0.396	0.019	0.855	0.197	0.210	0.007	0.971
LDL-C^a^	−0.050	0.740	−0.030	0.768	0.116	0.465	0.150	0.430
HDL-C^a^	−0.078	0.602	0.068	0.510	0.179	0.257	−0.095	0.618
Fasting plasma glucose^a^	−0.142	0.342	0.049	0.635	0.228	0.147	−0.160	0.398
Fasting insulin^a^	−0.119	0.426	−0.098	0.337	0.193	0.221	0.001	0.995
HOMA-IR^a^	−0.154	0.300	−0.101	0.327	0.260	0.096	−0.073	0.702
HbA1c^a^	−0.040	0.792	0.151	0.140	0.079	0.619	0.149	0.431
Duration of diabetes^a^	–	–	0.246	0.015	0.390	0.011	0.431	0.017
eGFR	0.156	0.294	−0.116	0.256	−0.374	0.015	−0.435	0.016
Urinary ACR	0.111	0.458	0.160	0.118	0.538	<0.001	0.502	0.005

a *P-value determined by partial correlation analysis (age, sex, and BMI adjusted)*.

**Table 3:** Multivariate regression analysis with ANGPTL8 as dependent variable.

	**Control subjects with NGT**		**Type 2 diabetic patients**					
			**A1**		**A2**		**A3**	
	**β**	***p***	**β**	***p***	**β**	***p***	**β**	***p***
SBP^a^	0.129	0.370	−0.111	0.280	−0.012	0.937	0.158	0.365
DBP^a^	0.144	0.327	−0.106	0.303	0.011	0.943	0.075	0.670
TG^a^	0.163	0.288	0.199	0.061	0.396	0.013	0.372	0.037
TC^a^	0.122	0.396	0.019	0.855	0.207	0.210	0.010	0.960
LDL-C^a^	−0.050	0.740	−0.032	0.768	0.119	0.465	0.171	0.397
HDL-C^a^	−0.079	0.602	0.067	0.510	0.184	0.257	−0.093	0.599
Fasting plasma glucose^a^	−0.146	0.342	0.049	0.635	0.230	0.147	−0.154	0.389
Fasting insulin^a^	−0.116	0.426	−0.100	0.337	0.198	0.221	0.001	0.995
HOMA-IR^a^	−0.153	0.300	−0.100	0.327	0.264	0.096	−0.069	0.695
HbA1c^a^	−0.039	0.792	0.148	0.140	0.077	0.619	0.136	0.457
Duration of diabetes^a^	–	–	0.247	0.015	0.391	0.011	0.396	0.018
eGFR	0.200	0.294	−0.128	0.256	−0.435	0.015	−0.453	0.016
Urinary ACR	0.107	0.458	0.160	0.118	0.538	<0.001	0.493	0.004

a *Age, sex, and BMI adjusted*.

### Mediation analysis

**Figure 1:**
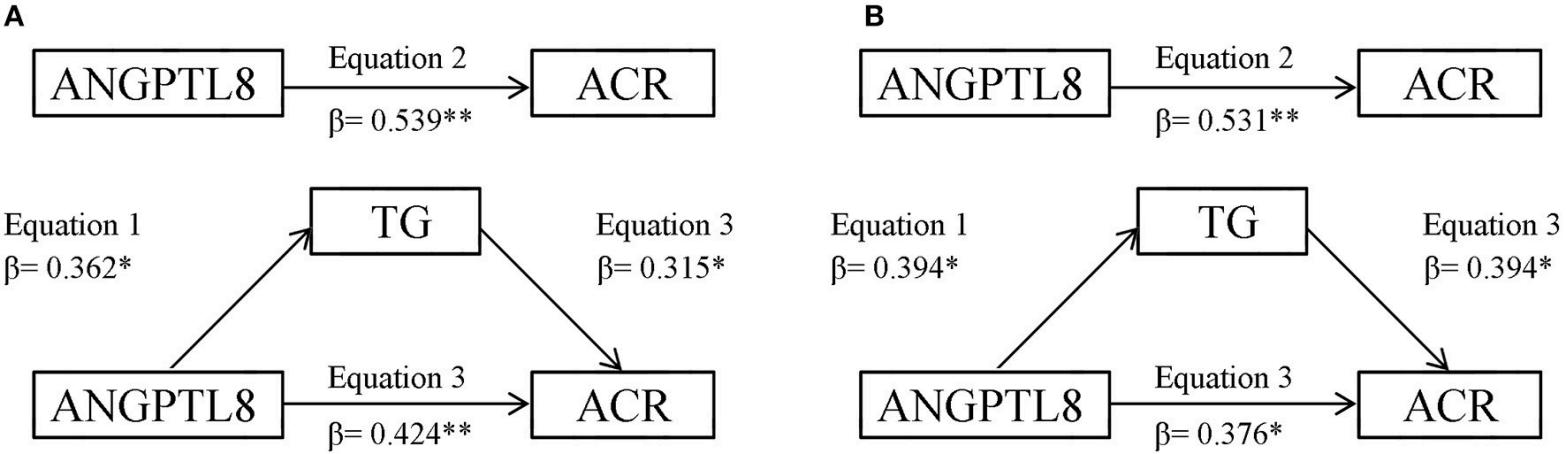
**(A)** TG mediation models of the relationship between ANGPTL8 and ACR in type 2 diabetic patients with A2 group. **(B)** TG mediation models of the relationship between ANGPTL8 and ACR in type 2 diabetic patients with A3 group. *P < 0.05, **P < 0.01.

Mediation analysis indicated that TG partially mediated the significant relationship between ANGPTL8 and ACR in both type 2 diabetic patients with A2 and A3 groups (Figure 1). When we tested the mediator role of TG in the relationship between ANGPTL8 and ACR in type 2 diabetic patients with A2 (Figure 1A), in the first linear regression equation, ANGPTL8 was positively associated with TG (*P* < 0.05). In the second equation, ANGPTL8 was positively associated with ACR (*P* < 0.001). In the third equation, when ANGPTL8 and TG were simultaneously included in the model, ANGPTL8 and TG were both positively associated with ACR (both *P* < 0.05). These data indicated that the effects of ANGPTL8 on ACR were partially mediated by TG. The percentage of total effect (direct plus indirect) mediated by TG was 21.2% (0.362^*^0.315/0.539). The analysis of the mediator role of TG in the relationship between ANGPTL8 and ACR in type 2 diabetic patients with A3 group showed similar results (Figure 1B), such that TG may be considered as partial mediator. The estimated percentage of total effect mediated by TG was 29.2% (0.394^*^0.394/0.531). Because HbA1c, fasting plasma glucose and HOMA-IR were not correlated with ANGPTL8 significantly, the mediator role of insulin resistance and hyperglycemia in the association between ANGPTL8 and ACR were not further evaluated.

### Associations between ANGPTL8 and albuminuria in type 2 diabetes

As presented in Table 4, multivariate binary logistic regression without adjustment demonstrated that serum ANGPTL8 levels were significantly related to A2 [odds ratio (OR) 2.49 (95% CI 1.17–5.32), *P* = 0.019] and A3 [4.51 (95% CI 2.01–10.12), *P* < 0.001] in type 2 diabetes. Following multiple adjustment with age, sex, and BMI, we observed that the OR for TG, duration of diabetes and ANGPTL8 were all significantly related to A2 and A3 (all *P* < 0.05), while ANGPTL8 had the highest OR and was significantly correlated with A2 [odds ratio (OR) 2.52 (95% CI 1.16–5.48), *P* = 0.019] and A3 [4.89 (95% CI 2.10–11.39), *P* < 0.001] in type 2 diabetes.

**Table 4:** OR (95% CI) by binary logistic regression models for albuminuria.

**Models**	**Covariates**	**T2DM with A1**		**T2DM with A2**		**T2DM with A3**	
		**OR (95% CI)**	***P***	**OR (95% CI)**	***P***	**OR (95% CI)**	***P***
Model 1	TG	1.0	–	1.47 (1.15, 1.87)	0.002	1.70 (1.28, 2.25)	<0.001
	Duration of diabetes	1.0	–	1.11 (1.01, 1.23)	0.030	1.15 (1.03, 1.28)	0.011
	ANGPTL8	1.0	–	2.49 (1.17, 5.32)	0.019	4.51 (2.01, 10.12)	<0.001
Model 2	TG	1.0	–	1.52 (1.18, 1.95)	0.001	1.71 (1.28, 2.28)	<0.001
	Duration of diabetes	1.0	–	1.12 (1.01, 1.23)	0.027	1.15 (1.03, 1.28)	0.013
	ANGPTL8	1.0	–	2.58 (1.20, 5.57)	0.016	4.44 (1.98, 9.96)	<0.001
Model 3	TG	1.0	–	1.46 (1.14, 1.87)	0.003	1.74 (1.31, 2.32)	<0.001
	Duration of diabetes	1.0	–	1.11 (1.01, 1.23)	0.032	1.15 (1.03, 1.28)	0.013
	ANGPTL8	1.0	–	2.45 (1.14, 5.23)	0.021	4.78 (2.09, 10.90)	<0.001
Model 4	TG	1.0	–	1.53 (1.18, 1.97)	0.001	1.70 (1.28, 2.25)	<0.001
	Duration of diabetes	1.0	–	1.11 (1.01, 1.23)	0.009	1.15 (1.03, 1.28)	0.012
	ANGPTL8	1.0	–	2.48 (1.16, 5.32)	0.019	4.73 (2.06, 10.86)	<0.001
Model 5	TG	1.0	–	1.57 (1.21, 2.05)	0.001	1.76 (1.31, 2.37)	<0.001
	Duration of diabetes	1.0	–	1.12 (1.01, 1.23)	0.028	1.15 (1.03, 1.28)	0.016
	ANGPTL8	1.0	–	2.52 (1.16, 5.48)	0.019	4.89 (2.10, 11.39)	<0.001

*Model 1 unadjusted, Model 2 age adjusted, Model 3 sex adjusted, Model 4 BMI adjusted, Model 5 age, sex, and BMI adjusted*.

## Discussion

The present study demonstrated that serum ANGPTL8 levels were significantly increased in type 2 diabetic patients with A1, A2, and A3 as compared with control subjects. In addition, the study proved a significant and positive relationship between serum levels of ANGPTL8 and ACR and identified TG as a partial mediator in this relationship. Furthermore, our data indicated that serum levels of ANGPTL8 had higher OR for albuminuria in type 2 diabetic patients, suggesting ANGPTL8 to be a new biomarker for albuminuria in type 2 diabetes.

Previous studies have investigated circulating levels of ANGPTL8 in type 2 diabetic patients; however, the results were conflicting (4). Some studies reported unchanged ANGPTL8 in type 2 diabetes, whereas others reported decreased or increased ANGPTL8 levels (4, 21, 22). The reasons for this discrepancy may be due to difference in study design, sample size, race, medicine status, handling of blood samples and use of ELISA kits. A recent meta-analysis of all published studies on ANGPTL8 and type 2 diabetes revealed increased circulating levels of ANGPTL8 in type 2 diabetic patients (15), consistently, our data demonstrated that serum levels of ANGPTL8 were significantly increased in type 2 diabetic patients as compared with control subjects, we also found that serum levels of ANGPTL8 in type 2 diabetic patients with A2 and A3 were significantly higher than in type 2 diabetic patients with A1. In addition, serum ANGPTL8 levels were found to be positively correlated with ACR and negatively correlated with eGFR in the subgroups of type 2 diabetic patients with A2 and A3. Due to the cross-sectional nature of our study, the causal relationship between ACR and ANGPTL8 cannot be elucidated, however, we still investigated this relationship by asking what factors might play an important role in mediating this relationship.

Hyperglycemia and insulin resistance have been considered as crucial risk factors for promoting the development of diabetic kidney disease (23). The relationships between glucose metabolism, insulin resistance and ANGPTL8 have been investigated in previous studies. In newly diagnosed type 2 diabetic patients, Hu et al found that serum ANGPTL8 positively correlated with fasting glucose and 2-h post-OGTT glucose (10), moreover, the results of their study and another research both proved that circulating ANGPTL8 levels were positively associated with indexes of insulin resistance in newly diagnosed type 2 diabetic patients (10, 11). However, in our study, no significant correlation was found between fasting plasma glucose, HOMA-IR and ANGPTL8 in treated type 2 diabetic patients, our results corresponded with the observation by Fenzl et al. (24). Therefore, it is speculated that antidiabetic medication might, at least in part, lead to this obscure relationship between insulin resistance, glucose metabolism and ANGPTL8 in type 2 diabetes. The mediating roles of hyperglycemia and insulin resistance in the relationship between ANGPTL8 and ACR were not evaluated because of these nonsignificant associations.

Aside from hyperglycemia and insulin resistance, lipid metabolism disorders have also been previously linked to the development of diabetic kidney disease (25). TG-rich lipoproteins can degrade glycocalyx, activate monocytes and increase permeability of the glomerular filtration barrier, all of which may contribute to the progression of diabetic kidney disease (25). Consistently, our results proved a positive correlation between TG and ACR. So far, mounting evidence from animal and human studies suggested that ANGPTL8 may play an important role in TG metabolism (10). Mice lacking ANGPTL8 had a significant reduction in plasma TG levels compared with control mice (5), whereas adenovirus-mediated overexpression of ANGPTL8 increased plasma TG levels more than 5-fold (6). Our current data and other epidemiological evidence in type 2 diabetic patients also demonstrated that serum ANGPTL8 levels were significantly and positively associated with TG (26). However, some other studies did not find this significant association in type 2 diabetes (10), the reasons for the inconsistence may be attributed to the fact that some participants in other studies might have been treated with TG-lowering drugs which would affect plasma TG levels, in this study, we excluded subjects with any use of TG -lowering drugs. The mediation analysis indicated that as ANGPTL8 increased, ACR increased, but when the influence of TG was controlled, the correlation between ANGPTL8 and ACR was mitigated, suggesting that TG might partially mediated this significant correlation. Consequently, one possible explanation for the positive correlation between ANGPTL8 and ACR is that ANGPTL8 might be involved in the pathogenesis of albuminuria in type 2 diabetes through increasing TG levels. However, given the cross-sectional design of the study, it is hard to conclude that TG mediates the impact of ANGPTL8 on albuminuria in type 2 diabetes. ANGPTL8 has also been suggested to play a role in cholesterol metabolism (24), however, our results proved that there was no significant relationship between ANGPTL8 and TC, LDL-C or HDL-C, we speculated this discrepancy might be partly due to the statin use in this study.

In our study, logistic regression analysis revealed that longer duration of diabetes, elevated levels of TG and ANGPTL8 were all significantly related to an increased risk of albuminuria in type 2 diabetes, in addition, ANGPTL8 had the highest OR for albuminuria risk among these parameters above, suggesting ANGPTL8 to be a novel biomarker for diabetic kidney disease in type 2 diabetes.

It is noted that there is a lack of association between ANGPTL8 and TG in controls subjects as well as age, however, two other studies have indicated a positive relationship between TG, age and ANGPTL8 in control groups (7, 27), in addition, one study found that ANGPTL8 was positively related to age but not TG (10). The reason for these discrepancies is unclear and probably due to the difference in sample collection (plasma versus serum), sample size, age groups and ethnicity. Future studies are needed to further elucidate this point.

Limitations of our study also deserve comments. First, our study is limited by the cross-sectional design and fails to address the causal relationship between albuminuria and ANGPTL8 in type 2 diabetes. Second, the association between ANGPTL8 and ACR might be related to more than a single mediator parameter (TG), further studies are necessary to determine more potential mediator variables. Third, the sample size of our study is relatively small (particularly in diabetic subgroups with A2 and A3) and it is lack of mechanistic basis, further studies are needed to confirm our findings. Fourth, our research is not the first one to show the involvement of ANGPTL8 in diabetic kidney disease development, previous study has already confirmed that ANGPTL8 is significantly increased in type 2 diabetic patients with different stages of albuminuria (26), however, in this study, we further highlighted the possible mediator role of TG in the relationship between ANGPTL8 and diabetic kidney disease in type 2 diabetic patients, this work might has some potential implications for future research investigating the underlying mechanism for this relationship.

In conclusion, we found that serum ANGPTL8 levels were significantly increased in type 2 diabetic patients with albuminuria, moreover, our data provided the evidence that ANGPTL8 was positively correlated with urine ACR in this population and TG might partially mediated this positive correlation. Our findings suggested of potential role of ANGPTL8 in the pathogenesis of albuminuria in type 2 diabetes.

## Author contributions

TZ, BG, HL, LY, and JS: contributed to the study concept and design. XZ, BG, JS, HL, LX, XH, HP, and LY contributed the data. TZ and LQ planned the statistical analysis. LQ conducted the statistical analysis. TZ, XZ and LY drafted the paper. All authors approved the final manuscript to be published and agreed to be accountable for all aspects of the work in ensuring that questions related to the accuracy or integrity of any part of the work are appropriately investigated and resolved.

### Conflict of interest statement

The authors declare that the research was conducted in the absence of any commercial or financial relationships that could be construed as a potential conflict of interest.
